# BEAT-IT: Comparing a behavioural activation treatment for depression in adults with intellectual disabilities with an attention control: study protocol for a randomised controlled trial

**DOI:** 10.1186/s13063-015-1103-5

**Published:** 2015-12-30

**Authors:** Andrew Jahoda, Craig Melville, Sally-Ann Cooper, Richard Hastings, Andrew Briggs, Dave Dagnan, Chris Hatton, Alex McConnachie, Chris Williams, Robert S. P. Jones

**Affiliations:** Institute of Health and Wellbeing, University of Glasgow, Academic Centre, Gartnavel Royal Hospital, 1055 Great Western Road, Glasgow, G12OXH UK; CEDAR, University of Warwick, Kirby Corner Road, Coventry, CV4 8UW UK; HETA, Institute of Health and Wellbeing, University of Glasgow, 1 Lilybank Gardens, Glasgow, G12 8RZ UK; Cumbria Partnership NHS Foundation Trust, Penrith CA11, Portland PI, UK; CeDR, Faculty of Health and Medicine, Furness College, Lancaster University, Lancaster, LA14YG UK; Robertson Centre, University of Glasgow, Boyd Orr Building, Glasgow, G12 ORR UK; School of Psychology, Bangor University, Brigantia Building, Bangor, Gwynedd, North Wales LL57 2DG UK

**Keywords:** Intellectual disabilities, Behavioural activation, Guided self-help, Psychological therapies, Depression, Randomised controlled trial

## Abstract

**Background:**

Depression appears to be more enduring amongst people with intellectual disabilities, suggesting that it is a more chronic problem or more poorly managed in this population. This is not helped by a lack of evidence about the effectiveness of psychological therapies for people who have intellectual disabilities and depression. Behavioural activation, which aims to counteract depression by increasing individuals’ level of meaningful activity and their exposure to positive reinforcers, has proven to be as effective as cognitive behavioural therapy in the general population. Given that this therapy makes fewer communicative demands and focuses on activity, it was thought that behavioural activation would be both accessible and apt for people with intellectual disabilities, who are often socially marginalised.

**Methods/Design:**

This study is a multi-centre single-blind randomised controlled trial of behavioural activation versus a self-help attention control intervention for depression in adults with mild/moderate intellectual disabilities. The study has an internal pilot in one centre, to establish that recruitment can be built up and sustained at the required level, before being rolled out across the other sites. One hundred sixty-six participants will be randomly assigned to the behavioural activation or self-help interventions, which will be delivered to individuals with mild to moderate intellectual disabilities, accompanied by someone who provides them with regular support. Both interventions are manualised and will be delivered over a period of approximately 4 months. The primary outcome measure will be the Glasgow Depression Scale, a self-report measure which is completed at baseline and 4 and 12 months post-randomisation. Secondary outcomes include measures of participants’ activity levels, proxy reports of depressive symptoms, and cost-effectiveness.

**Discussion:**

The study will provide evidence about the effectiveness of behavioural activation for depression, adapted for people who have mild/moderate intellectual disabilities, and will inform the delivery of psychological therapies to people with intellectual disabilities in practice.

**Trial registration:**

Date trial registered: Nov. 13, 2012; trial registration number: ISRCTN 09753005

## Background

Depression is highly prevalent amongst adults with intellectual disabilities. Studies suggest a point prevalence of depression of 5 % in adults with intellectual disabilities [1]. Depression is also more enduring when experienced by adults with intellectual disabilities than for the general population, suggesting that it is either a more severe disorder or more poorly managed. For example, a British cohort study found chronic depression to be five times more common in adults with intellectual disabilities compared with the general population [2]. Life circumstances may also contribute to the enduring nature of depression experienced by people with intellectual disabilities. For example, many individuals with intellectual disabilities experience social exclusion and a lack of meaningful or purposeful activity [3], factors associated with low mood.

The term ‘intellectual disability’ refers to people who have significant impairments of both intellectual and functional ability, with age of onset before adulthood. A significant proportion of the UK population has intellectual disabilities (also referred to as ‘learning disabilities’ in the UK). Approximately 2 % of adults and 3.5 % of children have an intelligence quotient of less than 70 [4, 5]. Individuals with intellectual disabilities have much higher levels of mental ill-health than the general population, with a point prevalence of 40 % for adults [6].

A great deal of effort has gone into developing and studying the efficacy of psycho-social interventions for depression in the general population. Yet such evidence is limited for people with intellectual disabilities and there is a need to redress this inequity. Recent efforts have focused on adapting cognitive behavioural treatment (CBT) models for use with individuals who have intellectual disabilities and several small controlled studies have produced promising results [7]. However, the effectiveness of CBT for depression still needs to be rigorously tested and CBT is not accessible for many individuals with intellectual disabilities, because of excessive cognitive and communicative demands [8–10]. The development of stepped care models has also demonstrated the value of having a range of evidence-based approaches available to fit with particular individual characteristics and circumstances [11].

A recent meta-analysis of studies with the general population found that behavioural activation is as effective as CBT in the management of depression [12]. The aim of behavioural activation interventions is to increase overt behaviours that are likely to bring the individual into contact with positive environmental contingencies, with a corresponding improvement in mood, thoughts, and overall well-being. Behavioural activation is less reliant than CBT on verbal communication and the ability of clients to talk about their emotions and thoughts. Therefore, for some adults with intellectual disabilities, behavioural activation may be more accessible and effective in the management of depression. Models of behavioural activation [13, 14] have evolved from earlier behavioural approaches and pay particular attention to the wider context of an individual’s life, and have a strong focus on understanding the function of behaviour. Establishing the function of behaviour for the individual is crucial because the aim is not merely to increase activity but to ensure that activities are purposeful and motivating for the individual. Taking account of the context of a person’s life may be especially important when working with marginalised and more dependent individuals.

An open trial of behavioural activation for people with intellectual disabilities and depression addressed a number of key feasibility questions, helping to provide the foundations for a large-scale trial [15]. Recruitment of people with intellectual disabilities and depression can be challenging, as people who have intellectual disabilities rarely refer themselves for psychological help with low mood. Moreover, their depressive symptoms can be masked by other emotional and inter-personal problems like anger or anxiety [16]. The findings from the open trial suggested that it is possible to identify and recruit people with intellectual disabilities and depression. The trial also found that the therapy was acceptable to the participants and there was excellent engagement with therapy and retention to 3 months’ follow-up. Finally, the self- and informant-report measures of depression proved to be sensitive to change.

An adapted manual of behavioural activation therapy for adults with intellectual disabilities was also produced for the open trial. The aim was to make the approach both accessible and relevant. In the first instance, the session materials and exercises were changed to reduce their complexity and to make them more engaging. The therapy was delivered to clients with intellectual disabilities alongside a support person who offers them regular help. People with intellectual disabilities may have limited opportunities to participate in a range of occupational or social activities [17]. By definition, they have problems with adaptive behaviour (day-to-day social, communication, and life skills) in addition to cognitive impairments [18]. This means they are likely to rely on a degree of support to take advantage of opportunities for activity that do arise. Hence, the first step to increasing their levels of activity is to ensure that the necessary opportunities and supports are in place. For a behavioural activation treatment to have ecological validity, in that it makes sense in the everyday context of the individuals’ lives, it is necessary to work alongside individuals’ families or paid carers, who are already providing them with help. This more systemic approach is also designed to improve the generalisation and maintenance of the intervention after therapy has finished. The third main change to the manual was to include a formulation to be shared with the client with intellectual disabilities and their support person. A first step toward effective joint working between the therapist, client and support person is to ensure that a shared understanding of the therapeutic process and objectives for therapy is achieved.

When the open trial was carried out, another key consideration concerned the potential to deliver the therapy in existing service settings. One of the challenges of implementing novel therapies is to ensure that there are sufficient numbers of therapists. Gaining access to psychological therapies can be a particular problem for people with intellectual disabilities. Hence, the aim was for the therapy to be delivered by a wider practitioner workforce, including community nursing.

The trial will evaluate the clinical and cost-effectiveness, compared with an attention control of guided self-help treatment, of a manualised behavioural activation intervention for adults with mild intellectual disabilities, in reducing self-report of depressive symptoms.

## Methods/Design

### Ethical and governance approval

Multi-centre approval has been granted by West of Scotland Research Ethics Committee 3. Research-and-development approval has been granted in all study sites: Cumbria Partnership National Health Service (NHS) Foundation Trust; Lancashire Care NHS Foundation Trust; Betsi Cadwaladr University Health Board; South Staffordshire and Shropshire NHS Foundation Trust; NHS Lanarkshire; NHS Ayrshire & Arran; and NHS Greater Glasgow and Clyde. The International Standard Randomised Controlled Trial Number (ISRCTN) reference number is ISRCTN 09753005.

### Design

**Fig. 1:**
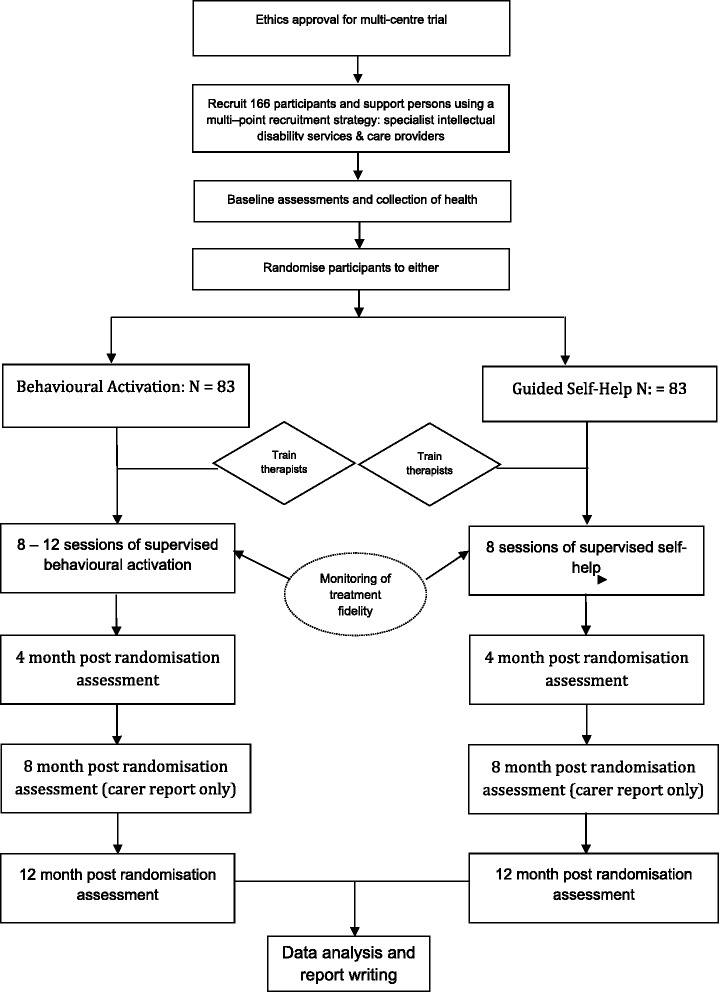
Flow diagram for the Beat-It study

This is a multi-centre randomised controlled trial (RCT) of behavioural activation compared with an attention control self-help intervention. It is a single-blinded design. Participants and therapists who conduct the therapy are not blinded, but the assessors of all measures are. The trial design is summarised in Fig. 1, and the particular behavioural activation intervention was given the acronym ‘Beat-It’. The guided self-help intervention was given the acronym ‘Step-up’.

In the first 18 months of the open trial of BEAT-IT, the mean (standard deviation, or SD) reduction in the Glasgow Depression Scale for People with Learning Disabilities (GDS-LD) [19] scores at 3-month post-intervention follow-up was 8.50 (5.24). We have powered the study to detect a mean between-group difference of 0.6 SD units, or 3.14 points on the GDS-LD. This makes the conservative assumption that the 4-month post-randomisation difference in score between the intervention and control groups will be 60 % of that observed from pre-test to follow-up in the open trial, thereby allowing for some improvement in the control arm of the trial, or for the observed improvement in the open trial being an overestimate of what will be achieved in a randomised trial, or for both. To have 90 % power to detect this effect size difference, the study requires 60 patients in each arm to provide outcome data at 12 months post-randomisation. The primary analysis will be an analysis of covariance adjusting for the baseline GDS score, which will have power to detect smaller intervention effects, depending on the level of correlation in scores over time.

There are no data to inform the effect of clustering of outcomes for patients seen by each therapist. Assuming that each therapist works with an average of nine participants (i.e., several part-time therapists at each site) and assuming an intraclass correlation of 0.025, the sample size must be increased by 20 % to 72 per group, or 144 in total. Recruitment of 166 participants will allow for up to 13.3 % loss to follow-up.

A meta-analysis of research with the general population [12] found a post-intervention effect size on self-reported depression symptoms of behavioural activation therapy versus supportive therapy of 0.75. These designs are the equivalent to our own attention control design. However, they did not report data regarding longer-term follow-up in comparison with supportive therapy. The effects relative to brief psychotherapy were 0.56 post-intervention and 0.50 after an average follow-up of 4 months, suggesting that the effects of behavioural activation therapy might persist for some time. Our follow-up of 12 months post-randomisation will be approximately 9 months post-intervention so we will be able to detect differences between groups only if they persist over a longer time frame than usually studied. Therefore, we believe that an effect size for sample size estimation purposes of 0.60 is realistic given the results of this meta-analysis for behavioural activation versus supportive therapy and would be considered to be of ‘moderate’ size and thus meaningful from a clinical perspective for an individual therapeutic intervention.

### Participant recruitment

A multi-point recruitment strategy [20] will be adopted, involving specialist intellectual disabilities services, relevant voluntary organisations, and social care provider organisations. Assistance will be sought from the mental health and primary care research networks at each of the centres, and recruitment strategies will include outreach work with voluntary provider organisations to help them to identify individuals with intellectual disabilities and depressive symptoms.

An internal pilot, to ensure that it is possible to recruit the target number of participants, is to be completed before the trial is rolled out across all sites. Therefore, recruitment will be completed over two phases.

*Phase 1*: Recruitment begins at the Scottish centre and is expected to be slow initially whilst contacts are established. It will also take time to educate potential referrers on appropriate patients to refer to the trial. However, recruitment should build up and be sustainable within 5 months.

*Phase 2*: If a minimum of 20 patients have been recruited at the end of the first phase or at least 16 patients recruited but with a recruitment rate of 4 per month in months 6 and 7, then the study will be rolled out in the other two centres in England and Wales. This will entail another 11 months of recruitment. Building on the experience from phase 1 in Scotland, recruitment should reach the same rate more rapidly in England and Wales. Therefore, we expect to recruit 166 patients into the study within 18 months of active recruitment. Potential participants are eligible for the trial if they meet all of the inclusion criteria and none of the exclusion criteria shown below.

#### Inclusion criteria

1. Mild/moderate intellectual disabilities, assessed by using the Wechsler Abbreviated Scale of Intelligence [21] and adaptive behaviour skills using a modified version of the Adaptive Behaviour Scale – RC2 [22].
2. At least 18 years old
3. Clinically significant unipolar depression using the Diagnostic Criteria for Psychiatric Disorders for use with Adults with Learning Disabilities [23].
4. Is able to give informed consent to participate
5. A level of expressive and receptive communication skill in English (reading skills not required) to allow participation in the treatment
6. Has a support person, a family member or paid carer who has supported them for ideally a minimum of 6 months to complete the screening and baseline visits OR is able to obtain information for the previous 4 months prior to randomisation. The carer, or another named individual, should be available for weekly-fortnightly treatment sessions with the practitioner and should currently provide a minimum of 2 hours support per week to the patient.

#### Exclusion criteria

1. Suicidal
2. A measured intelligence quotient of more than 75
3. Factors that prevent the participant from interacting with the carer and therapist or retaining information from the therapy (e.g., late-stage dementia, significant agitation, withdrawal arising from psychosis)
4. Does not consent to have her or his general practitioner contacted about their participation in the study.

#### Other participants

As stated in the inclusion criteria, a support person, who has known the participant for at least 4 months, will also be recruited to take part in the trial as an informant.

## Consent

Informed consent will be obtained from participants before any protocol-specific procedures are carried out. The decision of a participant to take part in the research will be based on a clear understanding of what is involved. Researchers employed on the study will be responsible for the process of seeking informed consent. The researchers will receive training on assessing capacity to consent, based on current UK legislation and established best practice.

Adults with intellectual disabilities are often supported by several carers. For example, they may have paid carers working shifts or different carers in their home and day placement environments. This means that the potential participant may not know whom they should discuss the study with. This can create a situation in which information sheets go missing before potential participants are able to discuss the study with carers and make an informed decision about whether they would like to participate. To take account of this, the member of staff who gives out the information sheet will be asked to notify a named person, who is independent of the research study, that an information sheet has been handed to a potential participant.

Participant information sheets for adults with intellectual disabilities use language appropriate to the developmental level of individuals with mild/moderate intellectual disabilities. A separate information sheet will be provided for family members and carers. Where an individual is interested in finding out more about the study, a researcher will arrange to meet with them to discuss the study and answer any questions they have. At the first meeting with the researcher, the participant will be given every opportunity to clarify points they do not understand and if necessary to ask for more information. The participant will be given sufficient time to consider the information sheets provided. It will be emphasised that the participant may withdraw their consent to participate at any time without loss of benefits to which they otherwise would be entitled.

Individuals who choose to take part in the research study will be asked to complete a written consent form. This will include providing consent to take part in interviews to express their views about the intervention. The consent form will be read through with the individual with intellectual disabilities and they will be asked to sign it, witnessed by a carer or another individual independent of the study. Those who do not have the capacity to consent to participation will be excluded from the research study.

### Randomisation

Participants will be allocated in a 1:1 ratio to one of the two study groups by using a blocked randomisation within each study centre; mixed block sizes of length 4 and 6 will be applied at random. Study centre and use of anti-depressants will be the two stratification variables.

Possible confounding factors will be carefully monitored, most notably participants’ use of (a) anti-depressants, St John’s Wort, and lithium and (b) other drugs which may have some mood-stabilising properties and are commonly prescribed in this population (an estimated 25 %, in view of comorbid epilepsy): carbamazepine, sodium vaporate, lamotrigine, and pindolol. Random allocation should ensure a balance of these two drug categories in the two arms. Changes in prescription over the duration of the study should be also be balanced in the two arms but we will also monitor this.

Participants will be randomly assigned by using an automated service maintained by the Robertson Centre for Biostatistics. All participants will be recruited and screened for suitability to participate in the study. Baseline data will also be collected before participants are randomly assigned.

## Trial procedures

### Treatments

The therapies will be delivered by specialist health-care workers for people with intellectual disabilities and general adult mental health workers, who will receive 2-day training in the therapy. The training consists of a background to the therapy models, the general principles of delivering psycho-social therapies and practice delivering key components of the therapy described in the manual. Each therapist was trained to deliver only one of the two interventions. In the main, it is anticipated that community intellectual disability nurses will be recruited to deliver the therapies. The therapists will also receive a minimum of fortnightly supervision by a clinical psychologist when they are delivering the therapy.

### Behavioural activation for depression (Beat-It)

The manualised therapy will be delivered to individuals alongside a supporter who provides regular support to them. There is an initial training session for carers regarding their role in the treatment and then 8–12 sessions held weekly to fortnightly, spanning approximately 4 months.

The treatment is divided into two phases, starting with an assessment period (four sessions), in which the participant and their supporter are socialised into the model and an individual formulation is developed. Key components of this phase include (i) identifying avoidant behaviours linked to depressive symptoms, and monitoring activity, (ii) identifying life goals, and (iii) psycho-education concerning the link between depression and activity. The assessment culminates in the presentation of a formulation to the participant and their carer (session 5). This provides a shared ‘story’ or common frame of reference for joint work between the participant, supporter, and therapist. Maximum participation by the person with intellectual disabilities is achieved by flexibly implementing the sessions in accordance with the treatment manual, and the particular approach taken is based upon the psychological formulation. The shared agreement of the carer regarding the treatment goals is also essential, as otherwise they are unlikely to be properly supportive of the intervention or willing to motivate the participants to achieve change.

The subsequent 5–10 active treatment sessions focus on (i) recovering lost skills and interests, and new skills training; (ii) graded exposure to reduce avoidant behaviours; (iii) targeting inherently reinforcing activity and activity likely to increase access to other positive reinforcers in three life domains: domestic tasks, purposeful daytime activity, and social/recreational activity; and (iv) addressing other emotional or inter-personal barriers to change, including anxiety and anger. The aim is for the sessions to be active and collaborative, with planned activities and role-play.

The final two sessions after the active treatment phase have a future focus and are concerned with helping the participant and supporter to maintain and build on progress they have made. A booklet is prepared for the participant and carer, reviewing progress and identifying changes that have been made, along with a plan for long-term maintenance and improvement. Completion of the treatment is defined as participation in a minimum of eight sessions.

### Guided self-help (attention control intervention)

The attention control intervention has been selected to be comparable to Beat-It in terms of carer and therapist attention, the use of a structured approach, and the support of a carer. Self-help materials were developed in Glasgow by Melville and colleagues [24] for use with adults who have intellectual disabilities and depression. The self-help resources were designed to be used by adults with intellectual disabilities along with the support of a carer. There will be an initial meeting with the participant and carer to explain the materials and provide coaching in their use and then eight sessions to support the participants and carers in their use of the self-help materials. Although the materials were designed to be accessible, carer support is essential for their delivery as the participants themselves are expected to have few, if any, literacy skills. The guided self-help model also meets the ethical criterion of being a meaningful comparison intervention in the absence of an alternative evidence-based treatment for people with intellectual disabilities who have depression. The participant and carer will be guided through a series of self-help materials by a therapist. The focus is psycho-educational, and the first two sessions with the participant begin by looking at the nature of depression before going on to outline how depressive symptoms can be tackled. The materials focus on key topics, including feeling down, sleep, exercise, and problem solving.

### Frequency and duration of follow-up

All participants will be followed up at 4 and 12 months post-randomisation. There will be one additional data collection point at 8 months with the carer alone, to chart any changes in the participant’s medication use and receipt of services.

### Outcome measures

#### Quantitative assessments

An overview of data collected at baseline and 4 and 12 months post-randomisation is shown in Table 1. Additionally, a purpose-specific questionnaire to gather demographic and health data about participants, and the participants’ expectations of therapy, will be completed at baseline only. Carers will also be telephoned at 8 months post-randomisation to complete the Client Service Receipt Inventory (CSRI).

**Table 1:** Outcome measure assessments

Outcome	Participant measures	Carer measures
Depressive and anxiety symptoms	Glasgow Depression Scale for People with Learning Disabilities (20 items: 10 minutes)	Intellectual Disabilities Depression Scale (38 items: 10 minutes)
	Glasgow Anxiety Scale-ID (20 items: 10 minutes)	
Aggression		The Behaviour Problems Inventory for Individuals with Intellectual Disabilities; Aggressive Behaviour sub-scale (10 items: 5 minutes)
Carer self-efficacy	Not applicable	Emotional Difficulties Self-Efficacy Scale (10 items: 5 minutes)
Participant-carer relationship	Not applicable	Expressed Emotion: Five-Minute Speech Sample
Activity measures and quality of life	EuroQual (EQ-5D 3 L; 5 items: 5 minutes)	EQ-5D 3 L (5 items: 5 minutes)
	Social Support Questionnaire (3 items: 10 minutes)	Modified Index of Community Involvement (46 items: 10 minutes)
		Modified Index of Domestic Participation (13 items: 5 minutes)
		ABS-RC:2 (4 sub-scales; 48 items; 10 minutes)
Response to life events	Bangor Life Events Schedule for Intellectual Disabilities (24 items: 10 minutes)	Not applicable
Health economics	EQ-5D (5 items: 5 minutes)	EQ-5D (5 items: 5 minutes)
		Client Service Receipt Inventory (30 items: 10 minutes)
		Medication inventory (10 items: 5 minutes)

### Quantitative outcome evaluation

#### Primary outcome measure

This will be a measure of depressive symptoms by using the Glasgow Depression Scale for People with Intellectual Disabilities (GDS-LD) [19]: This is a 20-item self-rating scale that requires the participant to indicate how often a particular symptom has occurred by using a 3-point scale (never/sometimes/always) during the previous week.

#### Secondary outcome measures

The Intellectual Disabilities Depression Scale (IDDS) [25] will provide an informant view of the clients’ depressive symptoms and will be completed by the carer. This is a checklist of behavioural symptoms of depression that can be observed by an informant. Other emotional and inter-personal difficulties that might be associated with depression will also be examined. Anxiety symptoms will be measured by using the Glasgow Anxiety Scale-ID (GAS-ID) [26], and aggressive behaviour will be measured by using the Behaviour Problems Inventory (BPI) [27].

Perceived and actual life changes that might be influenced by the interventions will also be examined, including Social Support using the Social Support Questionnaire (SSQ3) [28]. Changes in social and community-based activities will be measured by using the Index of Community Involvement (ICI) [29] and the Index of Participation in Domestic Life (IPDL) [30] aiming to capture changes in participation in household tasks. The Adaptive Behavior Scale - Residential and Community: Second Edition (ABS-RC2) [23] will be used as a proxy measure of avoidance of activity, a key aspect of behavioural change targeted by the behavioural activation treatment.

Patient-carer relationship will be assessed by using the Expressed Emotion: Five-Minute Speech Sample (FMSS) [31], and Carer Self-Efficacy will be examined by using the Emotional Difficulties Self-Efficacy Scale (EDSE) [32, 33]. This is a flexible scale to assess carer perceptions of their self-efficacy in specific support domains relating to adults with intellectual disability.

Finally, life events are recorded by using the Bangor Life Events Schedule for Intellectual Disabilities (BLESID) Self-Report [34] version. The BLESID will allow analysis of the potential changes in response to life events over time (e.g., reduced negative impact of new life events experienced during the course of the study), and recent exposure to life events (prior to screening assessment) will be included as a potential moderator of outcome in exploratory analyses. As this questionnaire has been developed to allow individuals to report on life events that have occurred over the last 12 months, it will be administered at baseline and 12-month follow-up only.

### Qualitative component

Up to 15 participants from each arm of the trial (n = 30) and 10 carers who attended therapy sessions in each arm of the trial (n = 20) will be interviewed. Participant and carer interviews will be guided by a semi-structured interview schedule. The questions will address the participants’ views and experiences of the Beat-It and Step-Up therapies in order to develop a better understanding of the change process. This part of the research is not hypothesis-driven. Instead, the main aim is to gain an ‘insider’s perspective’ that will assist with the interpretation of the quantitative results and help with the translation of the research findings into everyday practice. Focus groups of six study therapists from the Beat-It arm of the trial will also be held after all treatment is finished, to explore their experience of delivering the therapy and perceived barriers and successes.

#### Health economic evaluation

The economic analysis will be based on recorded health-related quality of life and health-care resources collected as part of the study. In keeping with UK guidance for conducting health economic evaluations, the EuroQoL instrument (EQ-5D Y) [35] will be used. A modified version of the EQ-5D Y will be used for the participants with intellectual disabilities. This version has more straightforward language aimed at young people who are at least 7 years old. However, the language is not “child-like” and so is appropriate for people with intellectual disabilities. The cost of delivery of the intervention itself will be based on timesheets that are collected as part of the study for the health service staff involved in the delivery of the intervention, costed at the mean of the relevant staff grade. The CSRI [36] will be used to record other health service contacts such as visits with community-based primary care, other health or social services, educational services, and outpatient and inpatient attendances. Unit costs will be taken from published UK reference costs. In combination with the CSRI, medication use will also be recorded and costed (Medication Inventory). Cost per quality-adjusted life-year of the BEAT-IT intervention will be calculated and results presented on the cost-effectiveness plane with statistical uncertainty represented by using confidence intervals or cost-effectiveness acceptability curves as appropriate.

#### Process evaluation

To establish fidelity to the Beat-It treatment, up to two randomly assigned sessions will be video- or audio-recorded with the permission of patients and their carers. One recording will be from the initial assessment phase (sessions 2–5) and a second from the active treatment phase (sessions 6–12). The recordings will be reviewed by an independent rater against a checklist of core requirements for (i) adherence to the manual, (ii) therapeutic process, and (iii) therapeutic alliance. The checklist has been adapted from an existing fidelity instrument, the Manualised Group Intervention Check [37], developed for group interventions and taking into account the particular social and communication skills required when working with people who have intellectual disabilities. Raters will be trained to a high level of initial reliability and then their ratings of session recordings will periodically be checked for drift.

Up to two recordings of the guided self-help attention control intervention, taken from initial sessions 2–5 and closing sessions 6–8, will also be reviewed as described above. The fidelity checks for the guided self-help intervention will cover the same non-specific elements of therapy as the Beat-It evaluation.

#### Data collection and blinding

The two treatments are of similar lengths and both involve a supporter meeting with the therapist alongside the patient. These similarities help to reduce the chance of the research assistants inadvertently finding out whether the patient has received behavioural activation or guided self-help, if patients make reference to their therapy in their meetings with the therapists. Other precautions include ensuring that none of the researchers carries out qualitative interviews with participants they are collecting data from or are involved with fidelity checks for them. Records will be kept of any instances of the researchers being unblinded and reported in research publications. The final statistical analysis plan will be subject to detailed review by a statistician who does not have access to the study data or randomisation schedule.

### Data analysis

#### Quantitative outcomes

The primary analysis will compare GDS-LD scores at 12 months post-randomisation between intervention groups, adjusting for baseline GDS-LD scores, study centre and use of anti-depressants at baseline within a mixed-effects linear regression model, including therapist as a random effect. Similar methods will be applied to the primary outcome measure at the immediate post-intervention assessment (4 months post-randomisation) and to secondary outcome measures at all assessment points. Repeated measures analysis, adjusting for stratification factors, will also be applied to each outcome measure. Models for the primary outcome will be extended to explore the effects of baseline characteristics, including the stratification factors, chronicity of depressive symptoms, life events, and history of previous failed psychological intervention. The moderating effects of these factors will be explored by using appropriately constructed interaction terms within linear regression models. These moderation analyses will be exploratory only and designed to inform future translation of the intervention into routine clinical practice. Similarly, we will also carry out exploratory analyses focused on potential mediation effects. In particular, changes to 4 months post-randomisation can be explored as mediators of effects to follow-up at 12 months post-randomisation, and therapist-rated session data can be used to explore potential mediators that may change within the therapeutic process.

### Qualitative outcomes

The interviews will be analysed by using framework analysis. This is a structured form of qualitative data analysis initially developed by the National Centre for Social Research [38] and designed for applied research. Rather than themes and sub-themes being wholly emergent from the data, framework analysis allows the researcher to start with a set of *a priori* themes which are used as an initial guide to the analysis, although in the analysis these themes can be altered and new themes can emerge from the data. Framework analysis is less labour-intensive than many other types of qualitative data analysis and allows for the systematic examination of data from relatively large samples for qualitative analysis.

For this study, the major *a priori* themes to begin the framework analysis will concern a number of dimensions that may inform the future uptake of intervention in clinical practice. These will include the following: participants’, carers’, and therapists’ perspectives on the process of change; helpful and unhelpful aspects of the interventions; factors relating to the three-way working relationship and the carer-patient relationship and barriers and facilitative factors relating to the maintenance of the interventions after cessation of contact with the therapist. Data from the focus group will be included in the framework analysis as additional evidence.

### Health economic outcomes

In the initial pilot stage, the contribution of the health economics element will be confined to ensuring that the appropriate data collection of resource use is made to support a full economic evaluation of the trial if the study proceeds to the second stage. The full economic analysis will compare the costs of the treatment with the quality-of-life benefits as measured by the EQ-5D in the 12-month post-randomisation follow-up period. The difference between treatment and control will be adjusted for any baseline differences in EQ-5D.

In addition, consideration will be given to potential cost-offsets associated with the treatment in terms of both direct costs to health and personal social services and direct costs to the patient and their carer [39]. The base case perspective will be the NHS and personal social services supplemented by a broader analysis that considers patient/carer costs. There are two components to the estimation of direct health service costs. The first is the costing of the treatments (behavioural activation therapy and the guided self-help) where the principal cost for these will relate to the time required to deliver the intervention. The second part of the costing will be to establish other service resource use and for that we will use the CSRI and the medication checklist. During the set-up phase, we will explore whether this format needs to be adapted for use with this population. The societal perspective will be assessed very simply as the time burden associated with participants and their carers attending for contacts with health/social services. Uncertainty in cost, quality-adjusted life-year (QALY) [40], and cost-per QALY estimates will be handled statistically through the use of non-parametric bootstrapping during the period of the trial. Extensive sensitivity analysis will be used to explore issues around whether unit costs might be somewhat different for patients with intellectual disabilities, the grade/salary of staff delivering the intervention and the number of sessions they can deliver per week, and the impact on cost-effectiveness of alternative assumptions concerning the durability of any treatment effect beyond the 12 months post-randomisation follow-up.

## Discussion

This is the first trial of behavioural activation for people with intellectual disabilities and depression and will be the largest trial of an individual psychological intervention for people with intellectual disabilities. There has been scepticism about the possibility of carrying out randomised controlled trials with this population, even though a recent review pointed to the paucity of robust evidence concerning the use of psycho-social interventions with people who have intellectual disabilities and mental health problems [41]. The best evidence is in relation to interventions for anger, one of the most common reasons that people with intellectual disabilities are referred for specialist psychological help. However, it is usually someone else who refers people with intellectual disabilities for help with an anger problem. This highlights a particular challenge with recruiting participants with a learning disability to a study of this nature: people rarely self-refer because they are experiencing depressive symptoms. Individuals’ distress may be expressed in different ways, and our recruitment strategy is to make it clear to those identifying potential participants that individuals with depressive symptoms may come to the attention of services because they are irritable, angry or anxious. Working out how to overcome barriers to recruitment is the reason for the internal pilot and a multipoint recruitment strategy. Successfully recruiting to this study could help to stimulate further trials of complex interventions for a broader range of mental health difficulties experienced by people with intellectual disabilities.

The active control arm is also a strength of the study, as comparing an intervention with treatment as usual fails to take account of a range of non-specific factors such as the therapeutic bond, which have been found to play a significant part in therapeutic change [42]. It could be argued that factors such as the therapeutic relationship and having an opportunity to be listened to take on an even greater significance in the lives of people with intellectual disabilities, who are often socially isolated and have limited social support. Hence, having a credible alternative intervention, with different active ingredients, should help to offer a clearer indication of behavioural activation’s contribution to change in depressive symptoms.

Even if proven psycho-social interventions are available, there remains the question of ensuring that people with intellectual disabilities gain access to such therapies. In this study, community nurses and other health professionals are trained and supervised to deliver the therapies. Investigating whether these professionals are able to deliver behavioural activation, whilst maintaining fidelity to the model, may be key to ensuring that services are able to deliver the therapy in routine clinical practice, where there is limited resource and expertise.

Careful thought has also gone into adapting behavioural activation for people with intellectual disabilities and depression. A crucial question is whether these changes mean that the intervention remains true to the behavioural activation model. Beyond monitoring therapist fidelity to the model, the secondary outcome measures should help to identify whether changes occur that are consistent with the model. For example, it will be possible to examine whether any improvements in depressive symptoms, the primary outcome measure, are associated with a change in activity.

Although the quantitative data will provide answers to the question of the effectiveness and cost-effectiveness of the intervention, they will not provide detailed insights into the process or mechanisms of change. However, initial data on this will emerge from the accounts of patients, carers, and therapists, about taking part in Beat-It and Step-Up. Data collected during therapy, including therapists’ logs of what happens in sessions, along with activity and mood data collected in sessions, will offer further insights into the change process or, indeed, why change does not occur. Interviewing participants and carers may also identify unanticipated outcomes of the therapy, either positive or negative. Finally, the qualitative data may provide insight into how the manualised approach can be used flexibly to accommodate different individual needs and circumstances, thereby assisting with the translation of the research findings into everyday practice.

## Abbreviations

ABS-RC2: Adaptive Behavior Scale - Residential and Community: Second Edition
Beat-It: Behavioural Activation
BLESID: Bangor Life Events Schedule for Intellectual Disabilities
BPI: Behaviour Problems Inventory
CBT: Cognitive Behavioural Therapy
CSRI: Client Service Receipt Inventory
DMC: Data Monitoring Committee
EDSE: Emotional Difficulties Self-Efficacy Scale
EQ-5D Y: EuroQoL
FMSS: Expressed Emotion: Five-Minute Speech Sample
GAS-ID: Glasgow Anxiety Scale
GDS-LD: Glasgow Depression Scale-Learning Disability
ICI: Index of Community Involvement
ID: Intellectual disabilities
IDDS: Intellectual Disabilities Depression Scale
IPDL: The Index of Participation in Domestic Life
QALY: Quality-adjusted life-year
SSQ3: Social Support Questionnaire
Step-Up: Guided self-help
RCT: Randomised controlled trial
TSC: Trial Steering Committee
